# Maternal Early Obstetric Warning System (MEOWS) as a Predictor of Maternal Morbidity and Mortality

**DOI:** 10.7759/cureus.104876

**Published:** 2026-03-08

**Authors:** Shikha Para, Shakti K Yadav, Prachiti Karode, Mukul Para

**Affiliations:** 1 Obstetrics and Gynecology, Gandhi Medical College, Bhopal, IND; 2 Pathology, All India Institute of Medical Sciences, Bhopal, Bhopal, IND; 3 Neonatology, Lokmanya Tilak Municipal Medical College, Sion, Mumbai, IND; 4 Gastroenterology, NIMS University, Jaipur, IND

**Keywords:** maternal early obstetric warning system, maternal morbidity, maternal mortality, obstetric screening, track-and-trigger system

## Abstract

Background

Maternal morbidity and mortality remain significant public health concerns, particularly in resource-limited settings. Early warning score systems, such as the Maternal Early Obstetric Warning System (MEOWS), have been recommended to facilitate the timely recognition of clinical deterioration in obstetric patients. This study evaluates the utility of MEOWS in predicting maternal morbidity and mortality in a tertiary care center in India.

Objectives

To determine whether MEOWS “track-and-trigger” charts can serve as an effective bedside screening tool to predict obstetric morbidity and mortality in women beyond 28 weeks of gestation through six weeks postpartum.

Methodology

A prospective observational study was conducted in the obstetrics department of a tertiary teaching hospital. A total of 500 women (≥28 weeks of gestation or up to six weeks postpartum) were monitored using the standard MEOWS chart. Vital signs and obstetric parameters were recorded at admission and regular intervals. A MEOWS “trigger” was defined as the occurrence of one red (severely abnormal) value or any two simultaneous yellow (moderately abnormal) values. Management followed standard hospital protocols without clinical interventions dictated solely by triggers. Maternal outcomes were classified as no morbidity versus morbidity/mortality by discharge. Statistical analysis included chi-square tests to compare characteristics and outcomes, alongside calculations of MEOWS performance metrics (sensitivity, specificity, predictive values) and relative risk (RR) for physiological parameters.

Results

Out of 500 patients, 93 (18.6%) triggered MEOWS, while 407 (81.4%) did not. The triggered group had significantly higher proportions of advanced age, grand multiparity, urban residence, referrals, and low socioeconomic status. On admission, 71% of triggered patients presented with obstetric complications compared to only 5.4% of non-triggered patients. Obstetric outcomes revealed a stark contrast: 37.6% of triggered women required emergency intervention versus 4.4% of the non-triggered group. All 15 maternal deaths occurred in the triggered group (16.1% case fatality). Overall, 76.3% of triggered women suffered severe morbidity or mortality, compared to only 2.0% of the non-triggered group. MEOWS demonstrated high diagnostic accuracy with a sensitivity of 76.3%, specificity of 98.0%, positive predictive value (PPV) of 89.9%, and negative predictive value (NPV) of 94.8%. A positive screen was associated with an approximate 38-fold higher risk of severe morbidity. Analysis of individual parameters indicated that tachypnea was the strongest single predictor of morbidity (RR: ~19.5), followed by diastolic hypertension (RR: ~15.4) and maternal tachycardia (RR: ~13.5).

Conclusion

In this prospective cohort, the MEOWS “track-and-trigger” system proved to be a valuable predictor of maternal morbidity and mortality. Women breaching trigger criteria were significantly more likely to experience severe complications or death. The MEOWS chart demonstrated high specificity and strong predictive value, correctly identifying the majority of women who developed obstetric morbidity with few false alarms. These findings support the incorporation of MEOWS into routine obstetric care to facilitate the early recognition of critical illness and improve maternal outcomes.

## Introduction

Maternal morbidity and mortality remain major challenges for public health, and maternal mortality is widely regarded as a key marker of the overall performance of a healthcare system. Maternal death has been defined by the World Health Organization as the death of a woman during pregnancy or within 42 days of termination of pregnancy, due to causes related to or aggravated by the pregnancy and its management [1]. In addition to mortality, a substantially larger number of women experience severe pregnancy-related complications, contributing to significant disease burden and preventable disability [1]. Despite improvements in maternal care, India continues to report a maternal mortality ratio of approximately 130 per 100,000 live births, with wide regional variation [2].

A critical aspect of preventing adverse outcomes is early identification of physiological deterioration. In many obstetric emergencies, abnormal vital signs and systemic warning symptoms occur before the onset of irreversible clinical collapse. However, the physiological changes of pregnancy may alter baseline parameters, which can delay clinical suspicion and timely escalation of care [3,4]. To address this, several early warning systems have been developed to support rapid bedside recognition of women at risk.

The Maternal Early Obstetric Warning System (MEOWS) is an obstetric-adapted “track-and-trigger” chart that incorporates pregnancy-specific physiological ranges. It uses routinely recorded clinical parameters to identify deviations requiring urgent review and intervention. It was developed as an obstetric-specific adaptation of the generic Early Warning Score (EWS), first recommended in the 2007 Confidential Enquiry into Maternal and Child Health (CEMACH) "Saving Mothers' Lives" report, following findings that early warning signs of impending maternal collapse went unrecognized in many reported deaths [3].

A MEOWS "trigger" is defined as: (i) one markedly abnormal "Red" parameter, indicating severe physiological compromise, or (ii) two simultaneously mildly abnormal "Yellow" parameters, indicating cumulative moderate risk. This color-coded "track-and-trigger" approach avoids numerical score summation, making it practical for rapid bedside application by nursing and junior medical staff, particularly in resource-limited settings. The following conditions are readily detectable by MEOWS: hemorrhage (hypotension + tachycardia), sepsis (fever + hypotension + tachycardia + hypoxia), venous thromboembolism (tachycardia + tachypnoea + hypoxia), pre-eclampsia/eclampsia (hypertension + hypoxia), and cardiovascular complications (tachycardia/bradycardia + hypoxia + hypotension) [5].

The CEMACH strongly recommended the use of MEOWS after reporting that missed warning signs contributed to maternal collapse in multiple cases [5]. Although MEOWS has been evaluated largely in high-income settings [6], evidence from low- and middle-income countries, where the burden, risk factors, and patterns of obstetric morbidity are distinct, remains limited.

Therefore, the present study was undertaken to evaluate the performance of the MEOWS chart as a bedside screening tool for predicting maternal morbidity and mortality in a tertiary care setting in northern India. The objective of the study was to evaluate the diagnostic accuracy (sensitivity, specificity, positive predictive value (PPV), negative predictive value (NPV), and overall accuracy) of the MEOWS "track-and-trigger" chart as a bedside screening tool for predicting obstetric morbidity, comprising hypertensive disorders, obstetric hemorrhage, sepsis/infection, shock, and pulmonary edema, and maternal mortality, in women at ≥28 weeks of gestation up to six weeks postpartum admitted to a tertiary care center in North India.

## Materials and methods

Study design and population

In this prospective cohort study, we utilized a consecutive enrollment process for eligible patients admitted to the obstetrics ward at Pt. B.D. Sharma Post Graduate Institute of Medical Sciences, Rohtak. Inclusion criteria comprised women at ≥28 weeks of gestation or up to six weeks postpartum who provided written informed consent. Exclusion criteria were strictly defined as: (i) gestational age <28 weeks, (ii) refusal of informed consent, (iii) inability to complete a baseline MEOWS assessment at admission (e.g., cases requiring immediate emergency surgery), and (iv) a hospital stay duration of <6 hours, which was deemed insufficient for meaningful clinical monitoring.

Study preparation

Prior to the study's commencement, all nursing staff and junior residents received standardized training on MEOWS chart completion and trigger criteria. To ensure consistency, a laminated MEOWS reference chart was displayed at each ward station. Adherence was monitored daily by the principal investigator, who reviewed completed charts for accuracy and completeness.

Data collection

At the time of admission, each participant underwent a detailed clinical evaluation, including history and general/obstetric examination. Vital signs and relevant obstetric parameters were systematically documented using the MEOWS chart adapted from the CEMACH (2003-2005) recommendations (Table 1) [3,7]. Recorded parameters included respiratory rate, oxygen saturation, pulse rate, systolic and diastolic blood pressure, temperature, neurological assessment (alert, verbal, pain, unresponsive (AVPU) scale), proteinuria, and assessment of lochia and liquor characteristics.

**Table 1:** Maternal Early Obstetric Warning System (MEOWS) chart parameters and trigger thresholds.

Parameters	Red trigger	Yellow trigger
Respiratory rate	<10 or >30 per min	21-30 per min
Oxygen saturation	<90	-
Heart rate	<30 or >120 per min	100-120 or 30-40 per min
Systolic blood pressure	<80 or >160 mm Hg	80-90 or 150-160 mm Hg
Diastolic blood pressure	>90 mm Hg	80-90 mm Hg
Lochia	Heavy/foul smell	-
Color of liquor	Green	-
Neuro response	Unresponsive, pain	-
General condition	-	Looks unwell
Temperature (°C)	<35 or >38	35-36

Physiological parameters were recorded on the MEOWS chart upon admission. Subsequent monitoring frequencies were standardized based on patient status: women admitted in labor were assessed every 12 hours until 24 hours postpartum, whereas puerperal admissions were monitored every four hours for the first 24 hours. Following these initial intervals, monitoring for all women was reduced to a once-daily frequency until hospital discharge. A MEOWS “trigger” was defined as either one markedly abnormal (red) parameter or the presence of two mildly abnormal (yellow) parameters recorded simultaneously. Importantly, clinical management and interventions were carried out as per routine institutional protocols and were not determined solely by MEOWS trigger status.

Outcome measures

The primary study outcome was maternal morbidity or mortality during the hospital stay. Maternal morbidity was classified using pre-defined criteria, including hypertensive disorders of pregnancy, eclampsia, obstetric hemorrhage, shock, suspected infection/sepsis, and pulmonary edema. At discharge, participants were categorized as (1) no morbidity or (2) morbidity/mortality.

Sample size and statistical analysis

The sample size was calculated based on a 57% prevalence of obstetric morbidity in India [2]. Using the formula, $n = \frac{Z^2pq}{L^2}$, where *p* = 0.57, *q* = 0.43, *Z* = 1.96, and an allowable error *L* = 10% of *p*, a minimum requirement of 317 patients was determined. Our final enrollment of 500 patients exceeded this threshold to enhance statistical power.

Data analysis was performed using SPSS version 23.0 (IBM Corp., Armonk, NY). Categorical variables were summarized as n (%). Group comparisons for demographic, clinical features, and maternal outcomes were performed using the chi-square test or Fisher’s exact test where applicable. The diagnostic performance of MEOWS was evaluated by calculating sensitivity, specificity, PPV, NPV, and overall accuracy. A p-value <0.05 was considered statistically significant.

## Results

Of the 500 women enrolled, 93 (18.6%) triggered the MEOWS chart, while 407 (81.4%) did not. Table 2 presents the parameters that had the highest red trigger frequency in our population.

**Table 2:** Frequency of the most common triggers in our study population.

Parameter	Yellow trigger, n (%)	Red trigger, n (%)
Diastolic blood pressure	17 (3.4%)	55 (11.0%)
Heart rate	23 (4.6%)	46 (9.2%)
Respiratory rate	45 (9.0%)	39 (7.8%)
Oxygen saturation	13 (2.6%)	23 (4.6%)
Neuro response	9 (1.8%)	27 (5.4%)
Proteinuria	0 (0.0%)	23 (4.6%)

Sociodemographic and clinical characteristics

As shown in Table 3, women in the triggered group were significantly more likely to be older (>26 years), reside in urban areas, belong to a lower socioeconomic class, and have a parity of three or more. The presence of pre-existing obstetric or medical conditions was also significantly higher in the triggered group (p < 0.00001). The most common presenting complaint in the triggered group was pre-eclampsia (47.0%), whereas anemia was the most common in the non-triggered group (54.5%).

**Table 3:** Comparison of key characteristics between triggered and non-triggered groups. Data are presented as n (%) unless specified. P-values were calculated using the chi-square test. Statistical significance is defined as p < 0.05.

Characteristics	Triggered (n = 93)	Non-triggered (n = 407)	Test statistic (χ²)	P-value
Age >26 years	37 (39.8%)	73 (17.9%)	20.95	0.000039
Urban residence	22 (23.7%)	27 (6.6%)	28.76	<0.00001
Low socioeconomic status	15 (16.1%)	10 (2.5%)	31.62	<0.00001
Parity ≥3	20 (21.5%)	49 (12.0%)	7.09	0.00759
Obstetric complaints present	66 (71.0%)	22 (5.4%)	190.76	<0.00001
Medical history present	27 (29.0%)	5 (1.2%)	90.07	<0.00001

Maternal and neonatal outcomes

Women who triggered the MEOWS chart had significantly worse outcomes (Table 4). They required a higher rate of intervention, including cesarean section and hysterectomy (37.6% vs. 4.4%, p < 0.00001). The duration of hospital stay was longer, with 28.0% of the triggered group staying for more than five days compared to 9.1% of the non-triggered group (p < 0.00001). Adverse neonatal outcomes, including intrauterine death, were significantly more frequent in the triggered group (13.0% vs. 0.2%, p < 0.0001).

**Table 4:** Comparison of outcomes between triggered and non-triggered groups. Data are presented as n (%) unless specified. P-values were calculated using the chi-square test. Statistical significance is defined as p < 0.05.

Outcome	Triggered (n = 93)	Non-triggered (n = 407)	Test statistic (χ²)	P-value
Intervention required	35 (37.6%)	18 (4.4%)	82.94	<0.00001
Hospital stay >5 days	26 (28.0%)	37 (9.1%)	25.65	<0.00001
Adverse neonatal outcome	12 (13.0%)	1 (0.2%)	52.03	<0.0001
Morbidity/mortality	71 (76.3%)	8 (2.0%)	259.73	<0.00001

Morbidity and mortality

A total of 79 women (15.8%) experienced significant morbidity or mortality. Of these, 71 were in the triggered group, and eight were in the non-triggered group. In the triggered group, 76.3% of women had morbidity/mortality, compared to only 2.0% in the non-triggered group (p < 0.00001). The most common morbidity was hypertensive disorders (28.0%), followed by obstetric hemorrhage (14.0%) and anemia (12.9%). There were 15 maternal deaths in the triggered group and none in the non-triggered group.

Table 5 describes the clinical characteristics, specific diagnoses, and MEOWS trigger profiles for the 15 maternal mortality cases in the study.

**Table 5:** Analysis of maternal deaths (n = 15). MEOWS: Maternal Early Obstetric Warning System; HELLP: hemolysis, elevated liver enzymes, and low platelet count syndrome; PPH: postpartum hemorrhage; Resp Rate: respiratory rate; HR: heart rate; BP: blood pressure; Neuro: neuro response; Temp: temperature.

Case No.	Age (years)	Diagnosis/Cause of death	Primary MEOWS triggers (red/yellow)	Hospital stay (days)	Clinical scenario & progression
1	24	Severe anemia/cardiac failure	Oxygen saturation, Resp Rate, HR, Neuro	5	Chronic anemia leading to cardiorespiratory collapse.
2	21	Hepatic failure (jaundice)	Oxygen saturation, Resp Rate, Temp, Neuro	15	Slow deterioration due to metabolic/hepatic failure.
3	26	Pulmonary tuberculosis/respiratory failure	Oxygen saturation, Resp Rate, BP, HR, Neuro	1	Acute respiratory failure upon admission.
4	28	Eclampsia/HELLP	Oxygen saturation, proteinuria, Resp Rate, BP, HR, Neuro	7	Hypertensive crisis with multi-organ involvement.
5	31	PPH/hemorrhagic shock	BP, HR, Neuro, general condition	1	Acute obstetric hemorrhage; rapid collapse.
6	23	Septic shock	Oxygen saturation, Resp Rate, HR, Neuro, Temp	1	Fulminant sepsis; rapid physiological trigger.
7	30	Ruptured uterus/shock	Oxygen Saturation, Resp Rate, HR, Neuro	1	Surgical emergency; rapid hemodynamic collapse.
8	24	PPH/hemorrhagic shock	Oxygen Saturation, Resp Rate, HR, Neuro	1	Acute hemorrhage post-delivery.
9	32	Severe anemia/heart failure	Resp Rate, HR, Neuro	1	Late presentation of severe anemia.
10	26	Hepatic encephalopathy	Proteinuria, Resp Rate, BP, HR, Neuro, Temp	5	Gradual neurological and hepatic decline.
11	32	Respiratory distress (unknown)	Oxygen saturation, Resp Rate, HR, Neuro	0	Sudden collapse/brought dead or immediate mortality.
12	20	Eclampsia	Oxygen saturation, Resp Rate, BP, HR, Neuro	2	Neurological collapse following hypertensive triggers.
13	24	PPH/shock	BP, HR, Neuro, general condition	0	Immediate postpartum collapse.
14	26	Sepsis/multi-organ failure	Oxygen saturation, Resp Rate, BP, HR, Neuro	7	Prolonged septic course with escalating triggers.
15	35	Septic shock	Oxygen saturation, Resp Rate, HR, Neuro, Temp	4	Infectious pathology with persistent red triggers.

Performance of the MEOWS chart

The MEOWS chart's performance as a screening tool for predicting obstetric morbidity and mortality is detailed in Table 6. The tool demonstrated a sensitivity of 76.34% and a high specificity of 98.03%. The PPV was 89.87%, and the NPV was 94.77%, with an overall accuracy of 94.00%.

**Table 6:** Performance of the MEOWS chart in predicting obstetric morbidity/mortality. Performance indices are presented as percentages (%). MEOWS: Maternal Early Obstetric Warning System.

Parameter	Value (%)
Sensitivity	76.34
Specificity	98.03
Positive predictive value (PPV)	89.87
Negative predictive value (NPV)	94.77
Accuracy	94.00

## Discussion

This study demonstrates that the MEOWS chart is an effective bedside tool for predicting maternal morbidity and mortality in a North Indian obstetric population. Nearly one in five women (18.6%) triggered the chart, and these women had significantly higher rates of adverse maternal and neonatal outcomes. The high specificity (98.03%) and NPV (94.77%) are particularly valuable, indicating that the tool can reliably identify patients who are not at immediate risk, thus preventing unnecessary interventions and resource allocation.

Our findings are consistent with previous research, although some differences exist. A UK-based study by Singh S et al. (2012) found MEOWS to have a higher sensitivity (89%) but lower specificity (79%) and PPV (39%) [6]. A subsequent study in India by Singh A et al. (2016) reported a sensitivity of 86.4% and specificity of 85.2% [7]. The higher specificity and PPV in our study may be attributed to the higher prevalence of severe morbidity and mortality in our patient population, which is typical of a tertiary referral center in a developing country. The leading causes of morbidity in our triggered cohort, hypertensive disorders and hemorrhage, align with established patterns of maternal morbidity in India [8].

The study also identified key risk factors for triggering the MEOWS chart, such as advanced maternal age, grand multiparity, and pre-existing medical conditions. This reinforces the need for heightened surveillance in these high-risk groups. The strong association between a MEOWS trigger and the need for intervention, adverse neonatal outcomes, and maternal death underscores its clinical utility. An abnormal physiological parameter is not just a number; it is an early warning of impending clinical deterioration. The relative risk analysis showed that a trigger in any parameter, particularly respiratory rate, heart rate, and blood pressure, significantly increased the risk of an adverse outcome.

The strength of this study lies in its prospective design and its application in a setting where such tools are most needed. By providing a simple, low-cost method for early risk stratification, MEOWS can empower healthcare providers to act proactively, potentially halting the progression from morbidity to mortality.

This study has certain limitations. First, it was conducted in a single tertiary referral center, which may limit generalizability to primary or secondary care settings, as being a tertiary government referral center, our facility receives a disproportionately high-risk case mix. Second, MEOWS parameters were recorded at defined intervals; therefore, transient physiological abnormalities between observations could have been missed. Third, management was as per standard protocols and not dictated solely by MEOWS triggers, and hence, the tool’s impact as an intervention strategy could not be evaluated. Larger multicenter studies are required to validate these findings and determine the feasibility of routine implementation across different levels of healthcare.

## Conclusions

The MEOWS chart is a valid, accurate, and highly specific bedside screening tool for predicting obstetric morbidity and mortality in a North Indian population. In this study, women breaching MEOWS trigger criteria were approximately 38 times more likely to experience adverse outcomes compared to non-triggered patients (76.3% vs. 2.0%). Notably, all 15 maternal deaths occurred exclusively within the triggered group. The tool’s high specificity (98.03%) and NPV (94.77%) are critical for minimizing "alarm fatigue" in resource-constrained environments. Respiratory rate abnormalities and diastolic hypertension emerged as the most significant individual predictors of risk. While these findings support the routine implementation of MEOWS to facilitate early recognition of at-risk women, it is important to note that the study demonstrates the tool's predictive utility rather than its interventional impact. The ultimate reduction in maternal mortality is contingent upon the speed and quality of the clinical response following a trigger. Future interventional studies are recommended to evaluate the efficacy of MEOWS-guided care pathways in improving maternal survival rates.
